# Proteomic-Based Insight into Malpighian Tubules of Silkworm *Bombyx mori*

**DOI:** 10.1371/journal.pone.0075731

**Published:** 2013-09-30

**Authors:** Xiao-wu Zhong, Yong Zou, Shi-ping Liu, Qi-ying Yi, Cui-mei Hu, Chen Wang, Qing-you Xia, Ping Zhao

**Affiliations:** State Key Laboratory of Silkworm Genome Biology (Southwest University), Chongqing, China; University of South Florida College of Medicine, United States of America

## Abstract

Malpighian tubules (MTs) are highly specific organs of arthropods (Insecta, Myriapoda and Arachnida) for excretion and osmoregulation. In order to highlight the important genes and pathways involved in multi-functions of MTs, we performed a systematic proteomic analysis of silkworm MTs in the present work. Totally, 1,367 proteins were identified by one-dimensional gel electrophoresis coupled with liquid chromatography-tandem mass spectrometry, and as well as by Trans Proteomic Pipeline (TPP) and Absolute protein expression (APEX) analyses. Forty-one proteins were further identified by two-dimensional gel electrophoresis. Some proteins were revealed to be significantly associated with various metabolic processes, organic solute transport, detoxification and innate immunity. Our results might lay a good foundation for future functional studies of MTs in silkworm and other lepidoptera.

## Introduction

Malpighian tubules (MTs) of insects were described by *Marcello Malpighi* in the seventeenth century, but were not functionally studied until the twentieth. The MTs extend from the junction of the midgut and hindgut into the abdominal body cavity [1]. MTs are excretory organs and function similarly to kidneys in higher animals [2], [3], eliminating waste products with water and maintaining a constant body composition despite changes in the external environment. The MTs of *Drosophila* perform organic solute transport, metabolism, and detoxification [4]. The adult tubules are critical in defense against insecticides, such as Dichlorodiphenyltrichloroethane, and against bacterial invasion [5]. It has been widely reported that the V-ATPase performed an important function in the tubules of *Drosophila* and mosquitoes [6], [7]. MT-specific genes in *Drosophila* are highly homologous to some disease-associated genes in humans [8], suggesting that the MTs can serve as organ models for the study of kidney diseases. Special functions have been revealed in some insects. For instance, the larvae of some beetles build protective cocoons using the silk materials excreted by MTs [9]. Leaf beetles retain a specialized set of MTs into adulthood and can create a sticky material for shield of their eggs [10].

The silkworm has three pairs of MTs, and two of them extend from two thirds of the midgut and finally insert into the rectum [11]. The MTs play critical roles in carrying out osmoregulation and excreting xenobiotics [12]. However, the details in their functions still remain largely unknown. Based on the genome data [13], [14], 5,073 active genes of MTs and 110 (2.17%) tissue-specific genes have been identified by microarray, which exhibit strong relevance to the physiological functions of MTs [15]. In recent years, the shotgun proteome technology becomes powerful in large-scale proteomic analyses. Various proteome profiles of silkworm tissues have been established covering head [16], integument [17], midgut [18], fat baby [19], embryos [20], peritrophic membrane [21], as well as some endocrine Organs [22]. In this study, Shotgun and 2-DE followed by MALDI-TOF MS were attempted to analyze the proteome profile of silkworm MTs from Day-5 fifth instar larvae. The raw data sets were searched against the in-house silkworm database with SEQUEST algorithms. To minimize the false positive sequence matches, the FDR (False discovery rate) of the identifications were searched through a target-decoy database and further validated by TPP and APEX analysis. A total of 1,367 proteins were identified in the MTs of silkworm; they are involved in ion and water transport, metabolism, detoxification and defense mechanism. This data may contribute to a better understanding of the function of silkworm MTs.

## Materials and Methods

### Experimental Animals and Sample Preparation

The Dazao strain (Chinese lineage, native inbred strain) was obtained from the Gene Resource Library of Domesticated Silkworm (Southwest University). The larvae were reared on fresh mulberry leaves at 26°C ±1°C under 75% ±2% relative humidity. The MTs were collected from Day-5 fifth instar larvae. The total MT protein was extracted in homogenization buffer (7 M Urea, 2 M Thiourea, 4% CHAPS, 0.2% Triton X-100, 50 mM DTT(Dithiothreitol), and 1% of a protease inhibitor cocktail) by tissue grinder. Protein concentration of the sample was determined by 2-D Quant Kit (GE Healthcare) according to the product specification.

### One-and Two-dimensional Gel Electrophoreses (1-DE and 2-DE)

1-DE for the MT protein sample was performed thrice by loading 1 mg of total MT protein on 12.5% SDS-PAGE gel. The gel was then stained with Coomassie Brilliant Blue R250 (Sigma), destained, and documented. Each sample lane was cut into 10 bands, and then diced into approximately 1 mm^2^ pieces. The pieces were trypsin-digested for LC-MS/MS using a linear ion trap mass spectrometer (Finnigan LTQ, Thermo Finnigan).

2-DE analysis was performed on 150 µg of total MT protein as previously described [23]. The protein sample was added to a 13 cm broad range IPG strip (pH 3 to 10 NL) (GE Healthcare) and diluted with rehydration solution (8.0 M urea, 2% CHAPS, 0.8% DTT, 0.5% IPG buffer, pH 3-10, and 0.002% bromophenol blue). Isoelectric focusing (IEF) was carried out by a gradient procedure: 50 V, 12 h; 100 V, 2 h; 200 V, 1 h; 500 V, 1 h; 1500 V, 0.5 h; 3000 V, 0.5 h; 5000 V, 0.5 h; 8000 V, 86000 Vhr. The current for each strip was limited to 50 µA. The strips were then immediately equilibrated for 15 min in buffer I (6 M urea, 50 mM Tris-HCl pH 8.8, 2% SDS, 30% glycerol, and 1% DTT) and re-equilibrated for another 15 min in buffer II, containing 2.5% iodoacetamide instead of DTT after IEF. 2-DE was performed in 12.5% polyacrylamide gel using Ettan DALTsix Electrophoresis System (GE Healthcare). The gel was stained with silver nitrate following the 2-DE [24]. Spots were scanned with a high-resolution image scanner (300 pixels/gel) and analyzed by ImageMaster 2D software (version 6.0, GE healthcare). The 2-DE experiment was replicated thrice.

### LC-MS/MS Analysis and Database Search

The gel pieces were bleached with 25 mM ammonium hydrocarbonate in 50% acetonitrile (ACN) and dehydrated with 100% ACN. The dehydrated gel pieces were incubated in adhesive block incubation A (50 mM Tris [2-carboxyethyl] phosphine (TCEP, Sigma) in 25 mM ammonium hydrocarbonate) for 1 h in the light and incubated for 0.5 h in the dark in solution B (100 mM iodoacetamide (GE Healthcare) in 25 mM ammonium hydrocarbonate). The proteins were digested overnight at 37°C in digestion buffer containing 20 ng/µl modified trypsin (Sigma). The resulting tryptic peptide mixture was extracted twice from the gel pieces with 5% trifluoroacetic acid (TFA) in 50% ACN solution and concentrated by vacuum centrifugation (LABCONCO). The evaporated extracts were resuspended with 0.1% methanoic acid (Sigma) and subjected to nanoLC-MS/MS using an Ettan MDLC nanoflow/capillary LC system (GE Healthcare). A constant flow rate of 200 µl/min was used for the nanocolumn with a 50 min solvent B (84% acetonitrile, 0.1% methanoic acid in water) gradient from 4% to 50%, and then from 60% to 95% in 10 min. The column was equilibrated with solvent A (0.1% formic acid in water) for 10 min before the next loading. The separated peptides were analyzed on the LTQ-Orbitrap mass spectrometer (Thermo) in positive ion mode. The capillary temperature was 200°C, and the spray voltage was 3.2 kV.

The local database was constructed based on the predicted 14,623 proteins [25](http://silkworm.swu.edu.cn/silkdb/doc/download.html). All MS/MS spectra were identified using TurboSEQUEST (BioWorksBrowser v.2.8, Thermo). The false-positive rate was estimated using the target-decoy database, a combination of forward and reverse protein sequences [26]. The search results were further validated using Trans Proteomic Pipeline (TPP, v4.2). The peptide and protein probability thresholds for running PeptideProphet and ProteinProphet were set at 0.9 and 0.95, respectively. Each identified protein was quantified by the APEX tool [27]–[29] using the information of identified peptides and theoretically identified peptides to estimate the relative protein abundances, and further normalized by the measured total protein concentration. Confidence was controlled by filtering the initial identifications to FDR ≤1% for each sample class.

### MALDI-TOF MS Analysis and Database Search

Protein spots were manually cut from the gel, and tryptic digestion was conducted as previously described [30]. Protein spots were excised and destained with 50 µL 30 mM potassium ferrocyanide and 50 µL 100 mM sodium thiosulfate. The pieces were washed twice with 100 µL milli-Q water and dehydrated with 100 µL 100% ACN. Then, 10 µL sequence-grade modified bovine trypsin (Sigma) (10 µg/mL in 25 mM ammonium carbonate) was added and incubated overnight at 37°C. The tryptic peptides were extracted twice with 50% ACN, in addition to 5% TFA, and concentrated to approximately 3 µL by vacuum centrifugation (LABCONCO). The tryptic peptides were equally mixed with a-cyano-4-hydroxycinnamic acid (Sigma) and placed onto sample plates. Mass spectrometry was performed on a Voyager DE PRO MALDI-TOF MS (Applied Biosystems) using delayed ion extraction and positive ion reflectron mode with an accelerating voltage of 20 kV, 60% to 65% grid voltage, and delay time of 100 ns. The autolytic peaks of trypsin were used for internal calibration. Mass spectral analysis and protein identification were performed according to a previous study [31]. The peptide mass fingerprintings (PMF) processed by Data Explorer software were searched against the local database (see above) by General Protein/Mass Analysis for Windows software (GPMAW, version 6.10).

### Bioinformatics

Gene Ontology (GO) annotation and analysis were performed using Gene Ontology (http://www.geneontology.org/) and WEGO (http://wego.genomics.org.cn/) as described by Ye et al. [32]. The EC numbers of the identified proteins were acquired (if available) with E-value ≤ e-10 using KEGG GENES BLASTP (http://blast.genome.jp/). The pathways in which at least three EC numbers were accepted were obtained from the KEGG reference pathway database (http://www.genome.jp/kegg/tool/search_pathway.html).

### Prokaryotic Expression and Preparation of Antiserum

The forward and reverse primers for amplifying cDNA sequence of *BmAGXT-2* (*BGIBMGA011600-TA*) were 5′-CCCATGGCATGCCCTCCACCGGGTTTACACC-3′ and 5′-CCGCTCGAGTCATTTCTTCGTGACCTTTTTAA-3′ (underlined restriction enzyme sites, Nco I and Xho I), respectively. The amplified fragments were subcloned into pET-29 expression vector. The recombinant plasmid was then transformed into *Escherichia coli* BL21 (DE3) strain. The recombinant proteins were induced by 1 mM IPTG for 4 h at 37°C and purified by incubating the supernatant with Ni-NTA Super-flow beads (Qiagen) according to the manufacturer's instruction. Polyclonal antibodies against BmAGXT-2 were produced by immunizing rabbit with purified recombinant proteins according to the traditional method.

### Western Blot Analysis

After 2-DE, the region (5 cm × 5 cm) containing BmAGXT 2 was cut and transferred to a PVDF membrane at a constant current of 300 mA at 4°C for 2.5 h. The blot was incubated overnight at 4°C in 5% skim milk in TBST (Tris-buffered saline, pH 8.0, and 0.1% Tween-20). The target proteins were detected by probing the blot with a primary antibody (anti-BmAGXT 2 antiserum, 1: 2,000) followed by a secondary goat anti-rabbit antibody conjugated to horseradish peroxidase (1: 20,000). The signals were detected by ECL advance Western Blotting Detection Reagents (GE Healthcare).

## Results

### Identification of 1,367 Proteins by 1-DE LC-MS/MS Analysis

**Figure 1:**
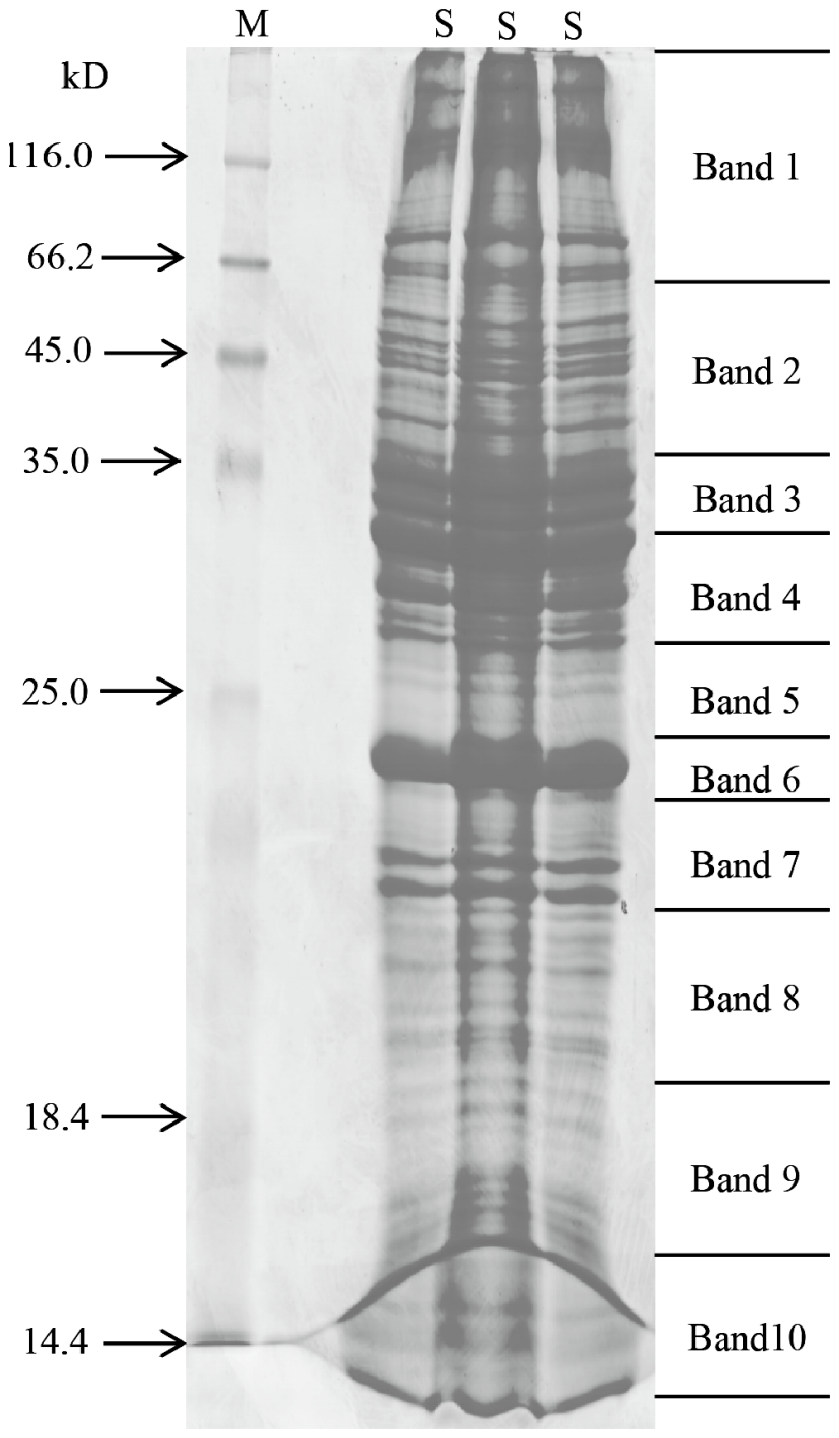
1-DE of the silkworm MTs. Lane M, molecular weight standards; Lane S, protein samples of silkworm MTs. Lane S was cut into 10 bands for the 1-DE LC-MS/MS analysis.

The MT samples were digested with trypsin, and the peptides were separated by 1-DE LC-MS/MS (Figure 1). The peptides identified from SEQUEST were further validated by TPP analysis. Moreover, the FDR of the identifications estimated by searching MS/MS spectra against a target-decoy database was 0.82%. In this study, each identified protein was quantified by a non-label quantitative proteomics, namely, APEX tool, which applies a correction factor (O_i_ value) for each protein that accounts for variable peptides detected by MS techniques. A total of 1,367 proteins were identified and listed in Table S1. Most of the identified acidic and basic proteins (97.82%) had isoelectric points (pI) of 3.82 and 12.92, respectively. The molecular weights of 80.54% of identified proteins range from 10 kDa to 100 kDa. Based on 1-DE gel analysis, the conserved hypothetical proteins (2,002.3 kDa) account for the largest part, whereas synaptosomal-associated protein (5.6 kDa) was the smallest.

### 2-DE MALDI-TOF MS Analysis and Western Blot Confirmed the Abundance of BmAGXT 2

**Figure 2:**
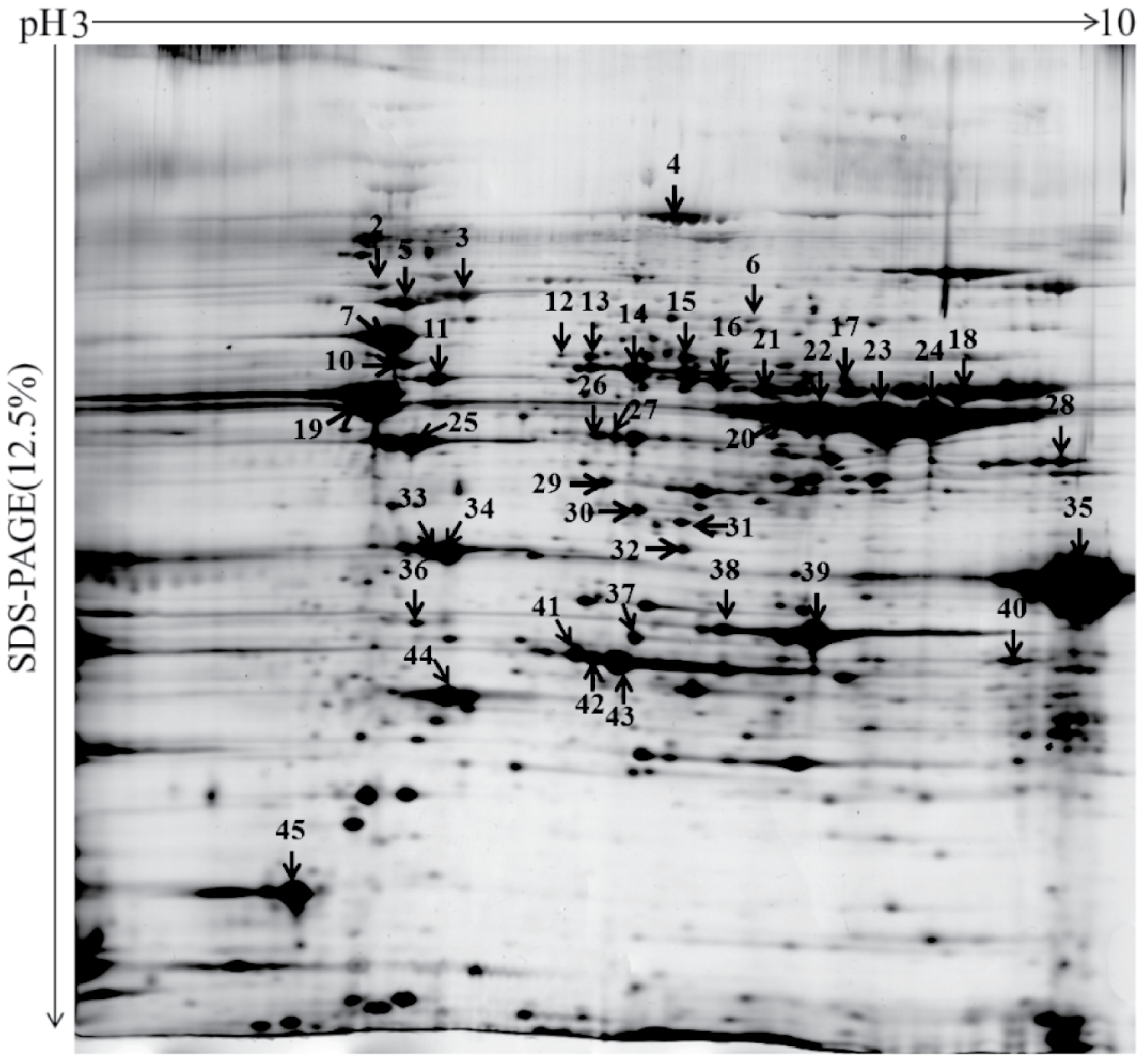
2-DE analysis of silkworm MT proteins. The protein samples were added to the pH-PAGE on a 12.5% polyacrilamide gel. The gels were stained using silver staining. Spots analyzed by MALDI-TOF MS were designated by numbers.

**Figure 3:**
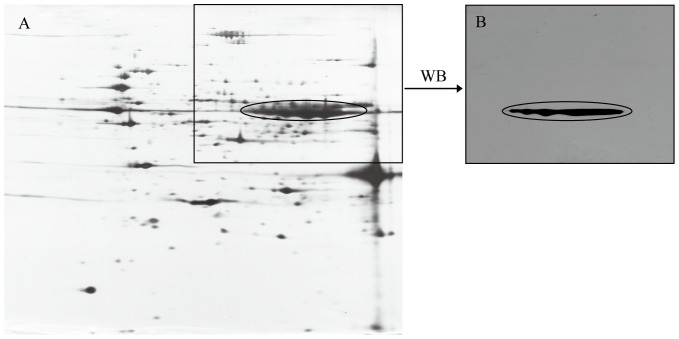
Target protein (BmAGXT 2) detected by Western blot analysis from a 2-DE map. (A). 2-DE map of silkworm MT proteins. (B). Western blot-detected BmAGXT 2 from MT protein samples.

The MT protein mixtures were separated on 2-DE gels using pH 3–10, 13 cm Readystrip IPG strip; the obtained maps were visualized by silver staining (Figure 2). Spots (450±8) were excised and digested, and then 45 unique proteins were identified by MALDI-TOF MS. Forty-one proteins were simultaneously identified by 2-DE and 1-DE LC-MS/MS (Tables S1 and S2). These proteins were highly abundant according to APEX analysis. A high abundance of BmAGXT 2 was also revealed by 2-DE analysis, and the observed shifts in its pI or molecular weight were probably caused by post-translational modification or protein degradation. Western blot analysis further showed the abundance of BmAGXT 2 in the MT samples (Figure 3).

### Gene Ontology Annotation and KEGG Pathway Analysis

**Figure 4:**
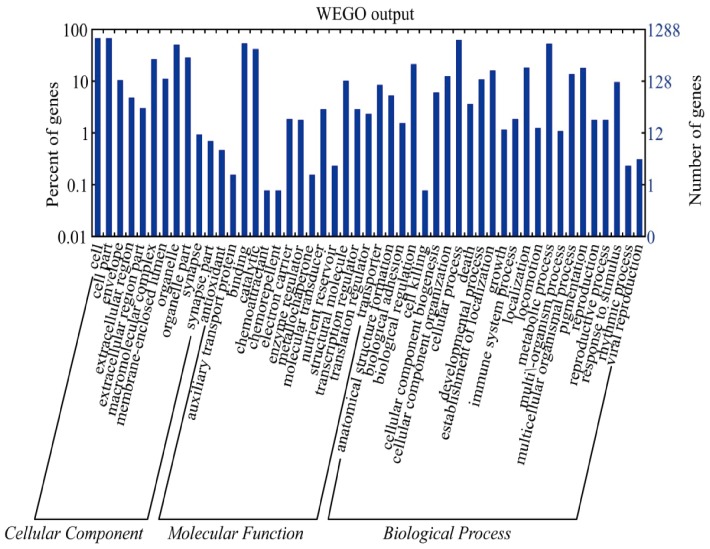
Distribution of the GO terms for all proteins identified from silkworm MTs. The identified proteins were classified into cellular component, molecular function, and biological process by WEGO.

Evidently, 1,288 out of the identified 1,367 proteins (1-DE LC-MS/MS analysis) were analyzed by homology-based GO annotation (E-value ≤ e-10). They could be divided into three categories, namely, cellular component, molecular function, and biological process (Figure 4). In the cellular component, the identified proteins are involved in different cellular processes, and the cell, cell part, organelle, and organelle part are mainly the members. From the perspective of molecular functional classification, the identified proteins in MTs are mainly related to binding and catalysis. The biological process category showed that the identified proteins are mainly involved in cellular processes, metabolic process, biological regulation.

**Figure 5:**
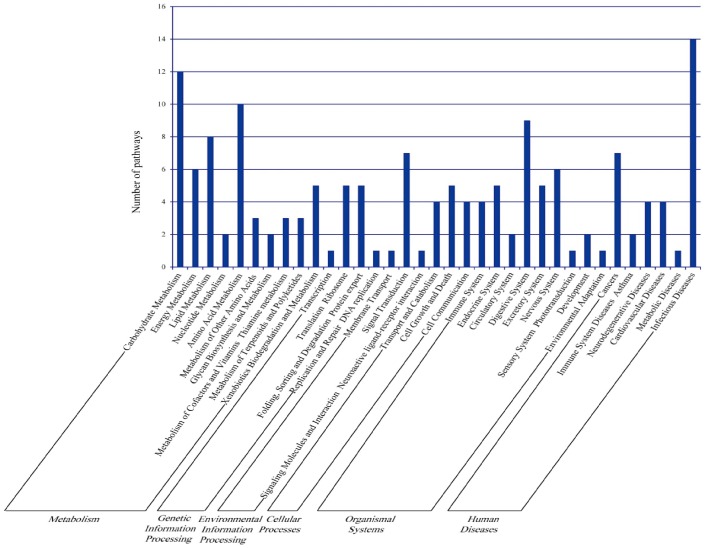
Categories of commonly related pathways in silkworm MT proteins according to KEGG pathway taxonomy. The pathways were methodically clustered into metabolism, genetic information processing, environmental information processing, cellular processes, and human diseases.

The proteins were subjected to analysis by KEGG tool, and 824 proteins were identified based on KO (E-value ≤ e-10). In order to increase the confidence of the identified pathways, only those with at least three ECs were further analyzed. All identified proteins were involved in 151 pathways, which were largely classified into metabolism, genetic information processing, environmental information processing, cellular processes, organismal systems, and human diseases (Figure 5). The results showed that the identified proteins involved in 151 pathways. As shown in Table S3, the most active pathway in MTs is metabolism. The common pathways of carbohydrate metabolism are more active than other metabolisms, but some pathways in metabolisms, such as the energy, lipid and amino acid metabolisms, in addition to xenobiotic biodegradation and metabolism, are also active in the MTs of Day-5 fifth instar silkworm larvae. The metabolism activities suggest that these MT organs may commit themselves to occurring much physiological and biochemical events at the feeding stage. Moreover, there are nine pathways related to the digestive system, indicating a close linkage relationship between the MTs and the digestive organs. In addition, four pathways are related to the immune system, which protected the MTs from infection. Several specific pathways, such as mTOR, TGF-beta, VEGF, Wnt, and MAPK signaling pathways, as well as ECM-receptor interaction, are closely interrelated with the kidney development and lesions of mammalian animals.

## Discussion

As we known, the methodology could be used to identify proteins using mass spectral data with EST dataset [33]. However, the ESTs are short, one-shot sequences with no overlapping sequencing and contain errors, and of course not as reliable as full genome sequences. In the present work, the proteome technology was attempted to characterize the silkworm larval MTs proteins profile. The raw data were searched against the local database which was constructed based on the predicted proteins from silkworm genome database with mass spectrometry software.Then, the FDR of the identifications were searched through a target-decoy database and further validated by TPP and APEX analysis. The approach presented in this study can minimize the false positive sequence matches, and provide clues for elucidating the functions of genes underlying specific processes and identifying candidate genes predicted to regulate traits of interest.

The mulberry silkworm, *Bombyx mori*, is a domesticated insect for silk production and a model lepidopteral insect for pest control. MTs of silkworm have important roles in excreting waste products and expelling toxins. Kajiwara et al. identified 127 proteins by MALDI mass spectrometry in silkworm MTs of fifth-instar day-3 larva [34]. Here in our work, a total of 1,367 proteins of silkworm MTs were identified. Our proteomics study will not only enrich proteomic data of silkworm MTs, but will also offer us an important insight into understand the role of MTs in silkworm. KEGG analysis showed that these MT proteins were involved in metabolism, genetic information processing, environmental information processing, cellular processes and organismal systems. Especially, cytochrome P450s and glutathione transferases are highly enriched in the MTs of silkworm. Cytochrome P450s comprise a large family of genes responsible for the oxidative metabolism which involved in the metabolism of pesticides principally in the insects. In *Drosophila*, cytochrome P450s exhibit tissue-specific expression pattern, manipulation of *Cyp6g1* in MTs could result in resistance to DDT and imperil the survival of the fly [35]. Glutathione transferase detoxifies both endogenous and xenobiotic compounds by conjugation reactions with reduced glutathione to produce endogenous and xenobiotic compounds more easily excreted by excretory organ, such as insect MTs [36]. McGettigan et al. found that MTs of *Drosophila* could sense bacterial challenge, and mount an effective killing response [37]. In this work, we identified two antimicrobial peptides from silkworm MTs, lysozyme and lectin, which are related to immune response. The nitric oxide synthase (NOS) produces nitric oxide (NO), an immune modulator in insects [38]. Although we could not identify the NOS from the tubule protein samples in this study, we found the NOS transcripts in the microarray data of silkworm MTs. To our knowledge, the midgut acts as the first line of defense against ingested xenobiotics, and the silkworm MTs might handle the detoxification metabolism of xenobiotics that appear in the hemocoel as *Drosophila.* Therefore, our data strongly indicated that the silkworm MTs might have analogous role to immune response.

Insects and humans diverged at least 400 mya, but very surprising similarities still exist in the functions of their genes. Chintapalli et al. found many *Drosophila* genes expressed in tissues analogous to those involved in human disease [39]. In this study, Vacuolar-type H^+^-transporting ATPase subunit B was successfully identified from 1-DE LC-MS/MS and 2-DE MALDI-TOF MS of silkworm MTs protein samples. V-type H^+^ ATPase plays major roles in proton transport and electrochemical gradients in eukaryotic cells. The disruption of V-ATPase subunits is not obligatorily lethal, but can result in the pH-dependent phenotype in yeast [40] and can influence *Drosophila* normal embryonic development [41]. In humans, V-type H+ ATPase mutations cause renal tubular acidosis and sensory deafness [42]. A xanthine dehydrogenase was identified from silkworm MTs and highly homologous to the rosy mutation (xanthine dehydrogenase) of *Drosophila.* The *rosy* mutation has the conserved symptoms of xanthinuria type I in humans [43]. BmAGXT-2 was identified by two different proteomic analysis methods. Western blot and activity analysis further revealed the presence of BmAGXT 2 in the MTs of silkworm. The drawback of *AGXT* leads to abnormal metabolism of oxalate, and causes a rare disease, primary hyperoxaluria (PH), which results in kidney stones even at an early age [44]. During growth and development, silkworm larvae accumulate massive amounts of calcium oxalate crystals in their MTs. This phenomenon indicates that the silkworm MTs might provide an ideal model system to study calcium oxalate crystallization in kidney.

Several proteins are also related to alcohol metabolism. Alcohol dehydrogenase encodes an alcohol oxidase, which plays an important role in the detoxification mechanism of alcohols. Alcohol metabolism occurs principally in the liver, where alcohol is first converted to acetaldehyde by alcohol dehydrogenase. Then, aldehyde dehydrogenase converts acetaldehyde to acetate. These two enzymes play a major role in metabolizing alcohol and in diminishing its effects in an organism [45]. In the MTs of silkworm, these enzymes might protect the silkworm from alcohol poisoning. 3-Hydroxyisobutyrate dehydrogenase (Hibadh) is a key metabonome enzyme that participates in valine metabolism and catalyzes the NAD^+^-dependent reversible oxidation to methylmalonate semialdehyde [46]. The high expression of Hibadh in the silkworm MTs implies that valine metabolism happens actively. We also found several organic solute transporters capable of excreting a huge majority of organic cations, anions, monocarboxylic acids, amino acids, and multivitamins. In summary, we revealed for the first time the protein profiles of silkworm MTs using shotgun proteomics. A total of 1,367 proteins were identified from silkworm MTs, hopefully laying a strong foundation for further study of special mechanisms involved in exreting metabolites and xenobiotics in the MTs of insects.

## Supporting Information

Table S1 **Identification of silkworm MTs proteins by APEX after 1-DE LC-MS/MS.** a) Protein accession numbers in silkDB and BGI numbers from silkworm genome. b and c) Protein description and classification derived from silkworm genome annotations in NCBI database. d and e) pI and Mr values of proteins obtained from ExPASy search results. f–i) False positive error rate (FPER), protein identification probability (pi), number of observed peptides (ni), and predicted number of proteotypic peptides (Oi) calculated by the APEX Quantitative Proteomics Tool. j) Relative abundance values calculated without estimated total number of protein molecules. * The proteins were simultaneously identified by 1-DE LC-MS/MS and 2-DE MALDI-TOF MS.
(XLS)

Table S2 **List of proteins identified from MTs of silkworm larva by 2-DE MALDI-TOF MS analysis.** * The proteins were simultaneously identified by 1-DE LC-MS/MS and 2-DE MALDI-TOF MS.
(XLS)

Table S3 **Detailed description of the KEGG pathway.**
(XLS)
